# Multiparametric Renal Magnetic Resonance Imaging for Prediction and Annual Monitoring of the Progression of Chronic Kidney Disease over Two Years

**DOI:** 10.3390/jcm12237282

**Published:** 2023-11-24

**Authors:** Charlotte E. Buchanan, Huda Mahmoud, Eleanor F. Cox, Benjamin L. Prestwich, Rebecca A. Noble, Nicholas M. Selby, Maarten W. Taal, Susan T. Francis

**Affiliations:** 1 Sir Peter Mansfield Imaging Centre, School of Physics and Astronomy, University of Nottingham, Nottingham NG7 2RD, UK; charlotte.buchanan@nottingham.ac.uk (C.E.B.); eleanor.cox@nottingham.ac.uk (E.F.C.); ppzbp@exmail.nottingham.ac.uk (B.L.P.); 2 Centre for Kidney Research and Innovation, University of Nottingham, Royal Derby Hospital Campus, Derby DE2 3DT, UK; huda.mahmoud@nhs.net (H.M.); msarn6@exmail.nottingham.ac.uk (R.A.N.); nicholas.selby@nottingham.ac.uk (N.M.S.);; 3 Walsall Healthcare NHS Trust, Manor Hospital Moat Road, Walsall WS2 9PS, UK; 4 NIHR Nottingham Biomedical Research Centre, Nottingham University Hospitals NHS Trust and the University of Nottingham, Nottingham NG7 2QW, UK

**Keywords:** chronic kidney disease, magnetic resonance imaging, multiparametric, progression, monitoring

## Abstract

Background: Multiparametric renal Magnetic Resonance Imaging (MRI) provides a non-invasive method to assess kidney structure and function, but longitudinal studies are limited. Methods: A total of 22 patients with CKD category G3-4 (estimated glomerular filtration rate (eGFR) 15–59 mL/min/1.73 m^2^) were recruited. Annual 3T multiparametric renal MRI scans were performed, comprising total kidney volume (TKV), longitudinal relaxation time (T_1_), apparent diffusion coefficient (ADC), Arterial Spin Labelling, and Blood Oxygen Level Dependent relaxation time (T_2_*), with 15 patients completing a Year 2 scan. CKD progression over 2 years was defined as eGFR_slope ≥ −5 mL/min/1.73 m^2^/year. Results: At baseline, T_1_ was higher (cortex *p* = 0.05, medulla *p* = 0.03) and cortex perfusion lower (*p* = 0.015) in participants with subsequent progression versus stable eGFR. A significant decrease in TKV and ADC and an increase in cortex T_1_ occurred in progressors at Year 1 and Year 2, with a significant decrease in perfusion in progressors only at Year 2. The only decline in the stable group was a reduction in TKV. There was no significant change in cortex or medulla T_2_* at Year 1 or Year 2 for progressors or stable participants. Conclusion: Lower renal cortex perfusion and higher T_1_ in the cortex and medulla may predict CKD progression, while renal cortex T_1_, TKV, and ADC may be useful to monitor progression. This study provides pilot data for future large-scale studies.

## 1. Introduction

Multiparametric renal Magnetic Resonance Imaging (MRI) shows great promise as a non-invasive method to assess kidney structure and function without exposure to radiation or gadolinium contrast agents [1,2]. This was highlighted in the COST Action PARENCHIMA [3], which initiated a drive towards standardisation of the renal MRI techniques of Blood Oxygen Level Dependent (BOLD) relaxation time or rate (T_2_* or R_2_*) [4,5,6], longitudinal relaxation time (T_1_) mapping [7,8,9], Arterial Spin Labeling (ASL) [10], and Diffusion Weighted Imaging (DWI) [11,12,13,14,15,16,17].

Chronic kidney disease (CKD) [18,19,20] is a heterogeneous condition, and there is a need for improved methods to stratify patients according to their risk of progression as well as to guide and monitor therapy. The development of renal multiparametric MRI for clinical or research use requires evidence that MRI parameters are useful to predict the progression of CKD and/or that MRI parameters change as CKD progresses, providing a method for monitoring progression and response to therapy. This will facilitate improved targeting of therapy to patients at greatest risk (personalised medicine) and more efficient trial design for the development of novel drug treatments.

To date, the majority of studies using renal MRI in persons with CKD have been cross-sectional analyses, confirming good reproducibility and reporting clear differences from healthy volunteers (HV), with CKD patients having higher T_1_ (reflecting increased inflammation and fibrosis), lower apparent diffusion coefficient (ADC) (reflecting molecular diffusion, which is decreased in the setting of fibrosis), lower cortical perfusion measured by ASL, as well as a moderate increase in R_2_* (corresponding to lower tissue oxygen) [21,22]. Renal MRI parameters have been evaluated against clinical measures of kidney function (glomerular filtration rate (GFR) and measures of proteinuria) and histological markers of fibrosis on kidney biopsy. The most consistent clinical and/or histological results associated with kidney damage in CKD have been shown with T_1_, ADC, and cortical ASL perfusion. The baseline data published from this longitudinal study by Buchanan et al. [21] showed that a model including cortical perfusion and cortical T_1_ predicted baseline estimated GFR (eGFR) (R = 0.87) and baseline log urine protein-creatinine ratio (log(PCR)) (R = 0.58), while a model including T_1_ and ADC predicted log(PCR) (R = 0.61). Berchtold et al. described a model including T1, ADC, and eGFR that predicted interstitial fibrosis (IF) with good discrimination (AUC 0.905 for ≥50% IF) [23].

A limited number of prospective studies have evaluated renal MRI to predict decline in kidney function, with fewer having collected serial MRI measures. In a BOLD-MRI study by Pruijm et al. [24] comprising 112 participants with CKD, the eGFR slope over 3 years was independently negatively associated with baseline 24 h urinary protein excretion and cortical R_2_*, and positively associated with the slope of R_2_* across the kidney parenchyma layers. In a study of 91 participants with CKD, multivariable analysis identified baseline T_2_*, eGFR, and PCR as independent predictors of eGFR slope over ~5 years [25]. In a post-hoc analysis of a randomised trial of phosphate binder and nicotinomide, baseline ADC in 122 participants with CKD was associated with eGFR slope over 12 months (*p* = 0.04) [26]. In a study of 24 subjects with moderate CKD who had annual BOLD, DWI, and ASL MRI measures over a 36-month period, Li et al. [27] showed that medullary R_2_* was the only MRI parameter independently associated with eGFR slope (*p* = 0.03). Further longitudinal studies are required to establish the interval required for serial MRI to monitor CKD progression or response to therapy.

In this pilot study, we collected serial clinical and MRI data at baseline, Year 1, and Year 2 in people with CKD. We aim to study whether baseline MRI measures can predict CKD progression and whether any MRI measures change over time to inform on their prognostic value and use for monitoring. The baseline MRI measures of this CKD group have previously been compared to healthy controls and correlated with baseline clinical measures in Buchanan et al. [21].

## 2. Materials and Methods

### 2.1. Participants

This study protocol was approved by the East Midlands Research Ethics Committee and registered at ClinicalTrials.gov (Identifier: NCT03578523). Participants gave written informed consent before they commenced the baseline MRI scan as published in Buchanan et al. [8] and consented to be contacted for follow-up MRI scans at Year 1 and Year 2. At baseline, participants were aged ≥ 18 years with CKD category G3-4 (eGFR 15–59 mL/min/1.73 m^2^ on at least two separate blood tests a minimum of 90 days apart). They were recruited from the outpatient nephrology patient population at the Royal Derby Hospital and had undergone a kidney biopsy as part of their clinical care within 90 days of enrollment. Exclusion criteria were an episode of acute kidney injury within the preceding three months, renal transplantation, contraindications to MRI, pregnancy, and the inability to provide fully informed consent.

### 2.2. Clinical Assessment and Protocol

Demographic data, medical history, and anthropomorphic measurements were assessed at each MRI study visit. This included blood pressure, eGFR calculated from serum creatinine concentration using the 2009 CKD Epidemiology Collaboration (CKD-EPI) [28,29] formula, and PCR measured on a single early morning urine sample. The progression of CKD was assessed for all 22 participants using the slope of all available eGFR values obtained from the electronic patient record during the 2-year observation period. A least squares regression method was used to calculate the eGFR slope over time, with a negative slope representing an annual reduction in eGFR [30,31,32]. Those with an eGFR decline of ≥5 mL/min/1.73 m^2^/year over two years were classified as CKD progressors, the others as stable. This classification for progressors is below the 25th percentile observed in a representative UK cohort of 967 participants with CKD for whom eGFR decline was −1.4 mL/min/1.73 m^2^/year (interquartile range −4.1 to 0.8) [33], and below the 95% confidence interval in a large meta-analysis of 66 trials of interventions in CKD for which the mean GFR decline in 186,312 participants was −3.17 mL/min/1.73 m^2^/year (95% confidence interval −3.61, −2.72) [34]. Renal biopsy tissue was collected at baseline and underwent standard histopathological processing and staining with Picro-sirius red solution to quantify the percentage of red staining reflecting collagen as a measure of cortical interstitial fibrosis.

### 2.3. Multiparametric Renal MRI

The multiparametric renal MRI scan protocol is described in detail in Buchanan et al. [21], scans were collected at each annual visit on a 3T Philips Ingenia MRI scanner. The acquisition and analysis are summarised below.

Balanced turbo field echo (bTFE) localiser scans were acquired in three orthogonal planes to quantify total kidney volume and plan placement of the five contiguous coronal oblique slices collected for multiparametric renal MRI. Total kidney volume was computed by manually tracing the kidney on the coronal bTFE localiser images (Analyze9^®^, AnalyzeDirect, Overland Park, KS, USA).

The following renal mapping data were all acquired with a 288 mm × 288 mm field-of-view, 3 mm × 3 mm in-plane resolution, 5 mm slice thickness, and were collected at end-expiration.

T_1_ mapping: Respiratory-triggered inversion recovery (IR) sequence with inversion times (TI) of 200/300/400/500/600/700/800/900/1000/1100/1200/1300/1500 ms with a fat-suppressed spin echo echo planar imaging (SE-EPI) readout (SENSE 2.3/TE = 27 ms, temporal slice spacing 58 ms). Data were fit to an inversion recovery curve to generate T_1_ and M0 maps.

Diffusion Weighted Imaging: Respiratory-triggered fat-suppressed SE-EPI DWI data (SENSE 2.3/TE = 67 ms) acquired at 11 b-values (0/5/10/20/30/50/100/200/300/400/500 s/mm^2^) in three orthogonal directions to reduce the influence of diffusion anisotropy. A maximum b-value of 500 s/mm^2^ was chosen to maintain an acceptable echo time and respiratory trigger. Apparent diffusion coefficient (ADC) maps were generated by fitting the log of the exponential signal decay to all b-values.

ASL perfusion: Respiratory-triggered Flow Alternating Inversion Recovery (FAIR) ASL data (selective(S)/non-selective (NS) thickness of 45/400 mm) were acquired with a SE-EPI readout (SENSE 2.3/TE 27 ms) with in-plane pre- and post-saturation pulses applied. ASL data were collected with post label delays of 1800 ms (25 S/NS pairs) and 300/500/700/900 ms (4 S/NS pairs) for inflow quantification, with a base M0 scan acquired. Perfusion S and NS data sets were realigned, and perfusion-weighted images were computed from subtraction of the NS from the S data and averaged to create a single perfusion-weighted (ΔM) map. ΔM, inflow, M0, and T_1_ maps were used in a kinetic model to calculate tissue perfusion maps.

T_2_* mapping: BOLD T_2_* data were acquired using a multi-echo fast-field-echo (mFFE) scheme (12 echoes, TE/∆TE 5/3 ms, SENSE 2, 25° flip angle, with 5 slices collected in three breath-holds). mFFE data were fit to form T_2_* maps from the log of the exponential signal decay.

Renal cortex and medulla definition and multiparametric MRI estimation: A histogram of T_1_ values within both kidneys was used to define a T_1_ threshold to segment renal cortex and medulla masks [2]. These masks were applied to the T_1_, ADC, and T_2_* maps, generating a histogram of each MRI measure for the cortex and medulla to which a Gaussian curve was fitted, and the mode and full-width-at-half-maximum (FWHM) were computed.

### 2.4. Statistical Analysis

Analysis was performed using SPSS version 29 (IBM©) and graphs generated using Prism 10 (GraphPad Software, Inc., version 10, La Jolla, CA, USA). A Shapiro-Wilk normality test was applied to each clinical and MRI measure. Normal data are expressed as mean ± standard deviation, and non-normal data as median (interquartile range, IQR). Since no significant difference in MRI measures was observed between the right and left kidneys (paired *t*-test), the mean measure across both kidneys was used in the analyses. Given the skewed distribution of PCR values, log(PCR) was used.

A Pearson correlation coefficient (normality test dependent) assessed the relationship between MRI and biochemical measures [eGFR and log(PCR)] across all baseline, Year 1 and Year 2 data points of both progressors and stable groups. Due to the small sample size, a multivariable analysis was not performed.

To determine baseline MRI and clinical measures that predict decline in kidney function, a *t*-test or Mann-Whitney U test (normality-dependent) was used to evaluate significant differences in the MRI measures between the progressor and stable groups at baseline. Receiver operating characteristic (ROC) curves were used to evaluate baseline measures to predict progression by measuring the ROC area under the curve (ROCAUC).

A Spearman correlation test of MRI and clinical measures to the eGFR slope was performed to determine indicators of CKD progression. To monitor CKD progression over time, the percentage change in each MRI measure from baseline was computed for Year 1 and Year 2 for subjects who completed all three MRI scans. Post-hoc pairwise comparisons of the percentage change were then conducted in the progressor and stable groups to determine which scan timepoints were significantly different from the baseline coefficient of variation (CV), and to determine differences between the progressor and stable groups at Year 1 and Year 2. Baseline CVs were defined from previously published short-term (<2 week) intra-subject between-session CVs of measures [2,21]. In addition, ROC curves were assessed to predict progression based on changes in measures from baseline to Year 1/Year 2.

## 3. Results

A total of 22 CKD participants were recruited for the baseline MRI scan, with 15 participants completing all three MRI scans (baseline, Year 1, and Year 2). Of the 22 participants scanned at baseline, 9 were classified as progressors and 13 as having stable CKD. A total of 18 participants returned for Year 1 visits (6 progressors, 13 stable), and 15 participants returned for Year 2 visits (5 progressors, 10 stable). Withdrawals were a result of two patients commencing dialysis, one patient developing lung cancer, and four patients declining to return. The demographics of the progressor and stable groups at each annual MRI visit are provided in Table 1.

### 3.1. Prognostic Value of the Baseline MRI and Clinical Measures

**Figure 1:**
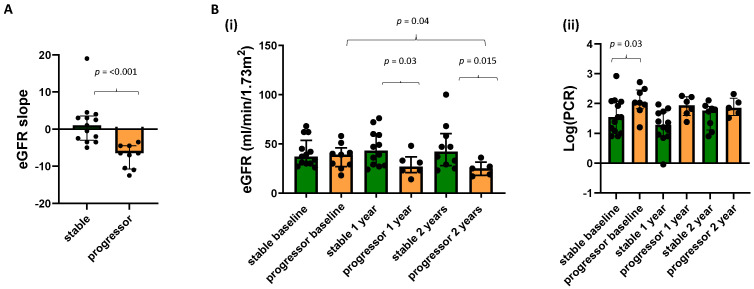
(**A**) eGFR slope between those participants classified as CKD progressors and stable, along with (**B**) (i) eGFR and (ii) log(PCR) at each annual MRI scan for the progressor and stable groups.

The prognostic value of the baseline MRI measures of TKV, T_1_, perfusion, ADC, and BOLD T_2_* was first investigated in the 22 CKD patients. Patients were divided into two groups of CKD progressors and stables according to eGFR slope (Figure 1A). Figure 1B shows the eGFR and log(PCR) collected at each annual MRI scan session for the progressor and stable groups. At baseline, there was no significant difference in eGFR measured between the progressors and stable group, but significant differences were seen at Year 1 and Year 2 timepoints, indicating adequate selection of progressors. For log(PCR), at baseline, a significant difference (*p* = 0.03) was seen between the progressor and stable CKD groups. No difference was seen in biopsy measures of fibrosis between the progressor and stable groups.

**Figure 2:**
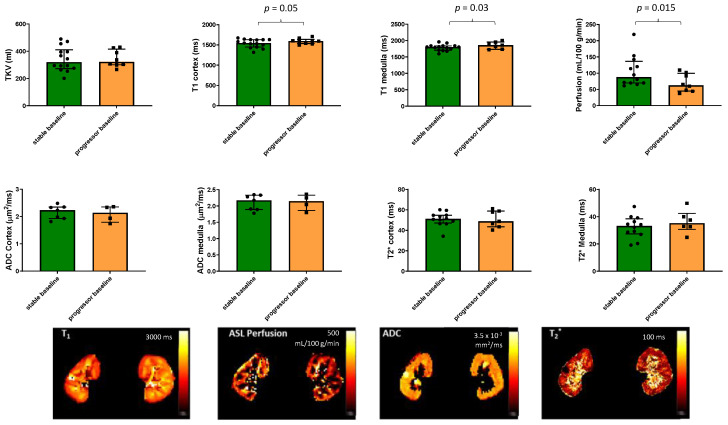
MRI measures of total kidney volume, T_1_, perfusion, ADC, and BOLD T_2_* in the 22 patients with CKD scanned at baseline for the progressor and stable groups. Significant differences between the groups are indicated and show a higher renal cortex and medulla T_1_ as well as lower renal cortex perfusion in the progressors compared to the stable group. Illustrative maps of each measure are also shown for the stable group.

Figure 2 shows the MRI measures at baseline for the progressor and stable groups. Progressors had a higher renal cortex T_1_ (*p* = 0.05) and medulla T_1_ (*p* = 0.03) as well as lower renal cortex perfusion (*p* = 0.015) than the stable group. Neither baseline TKV, cortex, or medulla BOLD T_2_* nor ADC values showed a significant difference between the progressor and stable groups.

Spearman’s correlation of baseline MRI and clinical parameters with eGFR slope showed a positive correlation with log(PCR) (R = 0.419, *p* = 0.053) and a significant negative correlation with perfusion (R = 0.382, *p* = 0.048). A ROC analysis of baseline measures between stable and progressor groups gave a significant result only for perfusion, which had a ROCAUC of 0.78 (95% CI 0.56, 0.96; *p* = 0.037).

### 3.2. Association between MRI Data and Biochemical Measures

**Figure 3:**
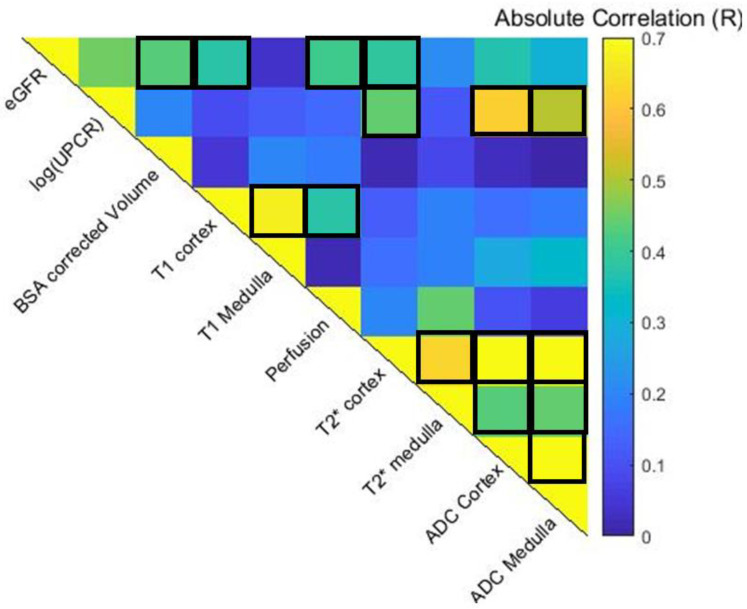
Spearman pair-wise correlation matrix of multiparametric MRI and clinical variables across baseline, Year 1 and Year 2 data points. Significant correlations with eGFR are seen for BSA corrected volume (*p* = 0.01), cortical measures of perfusion (*p* < 0.004), T1 (*p* = 0.008), T2* (*p* = 0.009), and ADC (*p* = 0.02). While ADC of the cortex (*p* < 0.0001) and medulla (*p* = 0.001) significantly correlated with the log(UPCR). Significant correlations were observed between some MRI measures. Positive correlations (*p* < 0.05) are outlined.

Figure 3 shows the pair-wise Pearson correlation matrix of the univariate analyses of each multi-parametric MRI and biochemical measure [eGFR and log(UPCR)] for all data points across baseline, Year 1, and Year 2 visits in the progressor and stable groups. BSA corrected volume (*p* = 0.01), cortical perfusion (*p* < 0.004), T_1_ (*p* = 0.008), T_2_* (*p* = 0.009), and ADC (*p* = 0.02) all correlated strongly with eGFR. While T_2_* of the cortex (*p* = 0.003), ADC of the cortex (*p* < 0.0001), and medulla (*p* = 0.001) significantly correlated with log(UPCR). Significant correlations were observed between some, but not all, MRI measures. As expected, there were significant correlations for individual MRI parameters between cortical and medullary measures. In addition, cortical T_1_ correlated with perfusion (*p* = 0.012) and cortical T_2_* with ADC (*p* < 0.0001).

### 3.3. Monitoring CKD Progression

**Figure 4:**
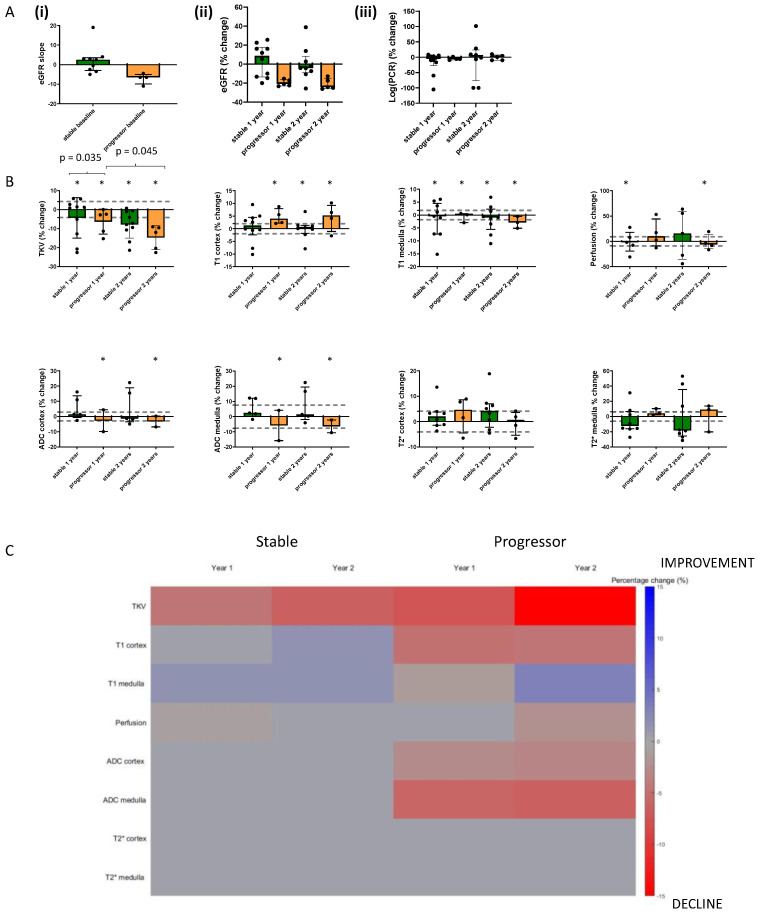
Bar charts showing the percentage change from baseline in (**A**) Clinical measures and (**B**) MRI measures at Year 1 and Year 2 separated into progressors and stable groups for those participants (*n* = 15) who completed all three MRI scan sessions. * indicates any significant difference from baseline at *p* < 0.05, and the values indicate any significant difference between percentage changes of groups or timepoints. Dashed lines indicate the coefficient of variance across time. (**C**) shows a heat map of those MRI values, which show a significant (*p* < 0.05) percentage change from baseline in MRI measures at Year 1 and Year 2 for the 15 subjects who completed all three MRI scans. Note that the heat map visualization shows the MRI measures that represent a decline in kidney function (i.e., reduction in TKV, reduction in ADC, reduction in BOLD T_2_*, and increase in T_1_), shown in red, and an improvement in blue. Any MRI measures with no significant change are shown in grey.

Figure 4A(i) shows the eGFR slope of the 15 participants who completed all MRI scan visits, while Figure 4A(ii) and (iii) show box plots of the percentage change from baseline in eGFR and log(PCR). Figure 4B shows bar charts of the percentage change from baseline in each MRI measure for the progressor and stable groups across each of the years. In the Supplementary Materials, these data are also provided to show all individuals (*n* = 18) who completed the Year 1 MRI scan. Figure 4C provides a heat map of MRI values shown in Figure 4B that showed a significant (*p* < 0.05) percentage change from baseline in MRI measures at Year 1 and Year 2 for the 15 subjects who completed all three MRI scans, divided into progressor and stable groups. The map shows the MRI measures in such a way that a change associated with decline in kidney function (i.e., reduction in TKV, reduction in ADC, reduction in T_2_*, and increase in T_1_) is shown in red, and conversely, a change associated with improvement is shown in blue. Any MRI measures with no significant change are shown in grey. More significant changes occurred in the progressors than in the stable group, with significant changes seen for perfusion, T_1_, ADC, and TKV. There was a significant reduction in TKV in the progressors and stable groups at both Year 1 and Year 2, but a significantly greater reduction (*p* = 0.035) in TKV from baseline to Year 2 in progressor versus stable groups. Also, TKV was significantly lower (*p* = 0.045) at Year 2 than Year 1 in the progressors. There was no significant change in renal cortex or medulla T_2_* at Year 1 or Year 2 for either the progressors or stable group. ROC analysis on the percentage changes in MRI measures from baseline to Year 1 between stable and progressor groups resulted in no ROCAUC of >0.75, whilst between baseline and Year 2 a ROCAUC of >0.75 resulted for TKV at 0.78 (95% CI 0.53, 1; *p* = 0.1) and 0.75 for T1 cortex at 0.75 (95% CI 0.41, 1; *p* = 0.16), for ADC cortex at 0.75 (95% CI 0.33, 1; *p* = 0.32) and ADC medulla at 0.83 (95% CI 0.51, 1; *p* = 0.18). but due to the small sample size, these did not reach significance.

## 4. Discussion

We show significant differences in baseline MRI parameters of renal cortex T_1_ and perfusion between persons who subsequently evidenced CKD progression and those with stable eGFR, suggesting that these MRI parameters may be useful to predict progression, with perfusion showing a significant ROCAUC between progressor and stable CKD, in addition to log(PCR), which is a well-evidenced clinical prognostic marker [25]. In contrast, the other MRI parameters of TKV, ADC, and T_2_*, as well as eGFR and the extent of interstitial fibrosis from biopsy, were not different between the stable and progressor groups at baseline in this study. Due to the small number of participants, it was not possible to perform a multivariate analysis and assess whether the observed differences in the baseline MRI parameters are independent predictors of CKD progression.

When combining all data points across all of the CKD participants, significant correlations were observed in MRI measures with eGFR, in particular for the renal cortex rather than the medulla (cortical perfusion, T_1_, and ADC). Correlations with protenuira were seen only in ADC measures, in agreement with Mao et al. [35] and T_2_* of the cortex.

During the two years of follow-up, TKV decreased across both stable and progressor groups, with this being more pronounced in the progressors, while cortical T_1_ increased and ADC decreased significantly in those with CKD progression but remained unchanged in those with stable eGFR. This suggests that TKV, cortical T_1_, and ADC MRI parameters may be best for monitoring progression, with all having plausible reasons to be sensitive to progressive fibrotic change. T_2_* did not change in association with eGFR decline over two years. Changes in the progressors at Year 1 were generally less or not significant, suggesting that the interval for serial MRI scans needs to be at least two years. Due to the small numbers, ROCAUC could not differentiate the groups but had values > 0.75 for TKV, cortex ADC, and T1 from baseline to Year 2. The percentage changes were assessed in the context of the between-session CoV over time, which provides an indication of the detectable reference change values as has been described. It should be noted that ASL perfusion has a larger intra-session CoV than the other MRI measures collected.

These results can be compared to the small number of prior longitudinal studies of renal MRI, which have mostly focused on renal BOLD MRI, for which we observed no significant predictions or changes with progression in this study. Prujm et al. [24] showed a higher cortical BOLD R_2_* and a reduced cortical to medullary R_2_* gradient at baseline in addition to proteinuria as independent predictors of the subsequent rate of decline in GFR. Interestingly, there was no change in R_2_* over 1 or 3 years of observation, though overall the rate of GFR decline was slow (−1.7 ± 7.7 mL/min/1.73 m^2^ at 1 year and −0.3 mL/min/1.73 m^2^ at 3 years). It should be noted that the study of Prujm et al. collected BOLD measures at a much higher spatial resolution (0.8 × 0.8 × 5 mm^3^ voxel size) than performed in this study, thus facilitating layer studies of the cortical medullary gradient.

In a retrospective study of 91 patients with CKD and a mean eGFR of 49 ± 29 mL/min/1.73 m^2^ at baseline [25], BOLD MRI and ADC were used to predict CKD progression. T_2_* was shown to be an independent predictor of the rate of GFR decline in addition to baseline eGFR and PCR, while ADC correlated with eGFR at baseline but was not a predictor of GFR decline. It should be noted that the mean decline in GFR over 5 years was also relatively small in this study, at −1.9 ± 3.0 mL/min/1.73 m^2^. Srivastasa et al. [26] performed post-hoc analysis of renal MRI scans in 122 participants with CKD from a randomised trial and reported that baseline R_2_* was not associated with eGFR slope, similar to our findings. They also reported that baseline ADC correlated with subsequent eGFR slope over 12 months, but this association was no longer significant after adjustment for albuminuria. No significant change was observed in R_2_* or ADC MRI parameters over 12 months in Srivastasa et al. In a study of moderate CKD [27], medullary R_2_* was the only baseline MRI parameter that independently predicted eGFR slope over 36 months. Medullary R_2_* and cortical ADC declined over time, but cortical perfusion did not change significantly, in keeping with the limited change seen in our results, except in our Year 2 progressor group.

In our study, higher baseline cortical T_1_ was significantly associated with subsequent GFR decline and increased over time in the group with CKD progression, suggesting that cortical T_1_ may be the most useful MRI parameter to both predict and monitor CKD progression. T_1_ measures are sensitive to changes in the water content of tissues and therefore increase with inflammation (cellular swelling and interstitial oedema) and fibrosis (collagen is associated with supersaturated hydrogel) [2]. Cross sectional studies have reported negative associations of T_1_ with eGFR and positive associations with proteinuria [21,22,23] as well as an association with the extent of fibrosis on kidney biopsies in some [8] but not all studies [22].

Lower renal cortical perfusion also predicted subsequent eGFR decline in this study (Figure 2), which is consistent with a large body of evidence that CKD progression is associated with loss of glomerular and peritubular capillaries. Renal ASL perfusion measures are not widely available and this measure has not been included in many previous clinical MRI studies. We have previously reported that in cross-sectional analyses, cortical perfusion was lower in people with CKD when compared with healthy volunteers, and among those with CKD, it was positively associated with eGFR and negatively associated with the magnitude of proteinuria and interstitial fibrosis [21]. We observed a significant change from baseline in perfusion only at Year 2 in the progressors but this may have been in part due to the relatively small number of participants with CKD progression who completed all scans (*n* = 5) and also the larger CoV for perfusion [2,21].

TKV was not different between the groups at baseline, likely reflecting a similar stage of CKD as indicated by eGFR and PCR being similar between the groups. Nevertheless, we did observe a significant reduction in TKV over time in both groups, with a greater decline observed in progressors, likely reflecting a loss of volume associated with fibrosis and CKD progression. Thus, TKV may represent a method for non-invasively monitoring progressive renal fibrosis, though further evidence is required to confirm this. This is potentially clinically useful because fibrosis may not initially result in a decline in GFR due to compensatory hyperfiltration by undamaged glomeruli. In this study, TKV measures were measured from a localizer; in future studies, higher-resolution T_2_-weighted scans from which TKV estimation can be automated using machine learning are recommended [36,37].

Results of ADC measures in cross sectional studies have been somewhat inconsistent but several have reported lower ADC in those with CKD versus healthy volunteers [21,22] and positive correlations with GFR [21,22,23,25,38]. One study reported a negative association between ADC and PCR [21], two have observed a negative association with fibrosis [21,23] and one has reported a negative association with inflammation [22]. In our study, there was no difference in ADC between groups at baseline; other studies have reported that lower ADC is associated with subsequent GFR decline [26]. We did, however, observe a significant reduction in ADC at Year 1 and Year 2 compared to baseline in both the stable and progressor groups, in line with the study of Li et al. [27] who previously reported a decline in ADC over 36 months associated with CKD progression. This suggests a potential clinical application for monitoring with ADC, if confirmed in larger studies.

Our study has several strengths. It is one of a small number to report the results of serial scans to simultaneously investigate the clinical value of multiple MRI parameters. However, some limitations should be considered in interpreting our findings. We had a small sample size and studied 22 participants, only 15 of whom completed all three MRI scans, so our analysis may have lacked statistical power to detect less robust associations. Nevertheless, the data have been the basis for the design of the AFiRM study (Application of Functional Renal MRI to Improve Assessment of CKD), a larger, multicenter study with a 2-year follow-up that is due to complete baseline recruitment in 2023 [39]. Further, the MRI acquisition measures that were used in this current study were kept consistent with the baseline MRI scans. Since this time, there has been rapid development in improved MRI acquisition and analysis methods. Future studies should use improved spatial resolution BOLD T_2_*/R_2_* measures for improved assessment of the cortex and medulla. The T_1_ mapping data collected in this study had limited spatial resolution, affecting the segmentation of the medulla and cortex; this may have confounded the accuracy of the medulla T_1_ measures. In future studies, machine learning methods can now be applied for image segmentation to generate more accurate TKV, cortex, and medulla masks. In the future, improved harmonised protocols such as those used in the UKRIN-MAPS (UK Renal Imaging Network: MRI Acquisition and Processing Standardisation) protocol [40] could be used for assessment of the parameters highlighted here, along with other measures including renal flow, the magnetization transfer ratio (MTR), and chemical exchange saturation transfer (CEST), which are expected to provide important evidence to support the clinical application of renal MRI.

## 5. Conclusions

Our results suggest that lower renal cortex perfusion and higher renal cortex and medulla T_1_ may be predictors of CKD progression, whereas cortex T_1_, TKV, and ADC may be useful MRI measures to monitor progression. Repeat scanning after a 2-year interval may be better for monitoring than at 1 year, which revealed fewer changes in MRI parameters. Further studies are required to confirm these findings in a larger cohort of patients before renal MRI can be recommended for routine clinical use.

## Figures and Tables

**Table 1:** The demographics of the progressor and stable groups at each annual MRI visit (baseline, Year 1, and Year 2). * indicates a significant difference (*p* < 0.05) between the progressor and stable group at a given timepoint; note that statistics are computed for log(PCR). Note a significant difference in proteinuria between the progressor and stable participants at baseline, but at Year 1 and Year 2, due to withdrawals, the protenuira of the progressor group was not as high, resulting in no significant difference between groups.

	Baseline		Year 1		Year 2	
	Progressors (*n* = 9)	Stable (*n* = 13)	Progressors (*n* = 6)	Stable (*n* = 12)	Progressors (*n* = 5)	Stable (*n* = 10)
Ethnicity (no. Caucasian]	7	11	4	10	4	8
Gender (no. male)	7	10	4	10	4	7
Age (years)	58 ± 16	57 ± 18	62 ± 38	59 ± 17	58 ± 19	60 ± 13
Height (m)	174 ± 9	274 ± 9	171 ± 7	173 ± 9	174 ± 7	172 ± 9
Weight (kg)	85 ± 11	89 ± 14	90 ± 16	87 ± 11	88 ± 16	88(20)
BMl (kg/m^2^)	28 ± 3	30 ± 6	29 ± 4	29 ± 4	29 ± 3	28 ± 3
Serum creatinine (umol/L)	160 ± 37	176 ± 37	207 ± 44	150 ± 39 *	231 ± 28	149 ± 47 *
eGFR (mL/min/1.73 m^2^)	37(13)	41 ± 14	29 + 12	46 + 18 *	25 ± 7	47 ± 24 *
Urine PCR (mg/mmol)	160 + 168	35(107) *	100 ± 64	30 ± 29	94 ± 70	55 ± 41
Systolic blood pressure (mmHg)	142 + 17	136 ± 18	132 ± 11	140 ± 18	140 ± 20	142 ± 15
Diastolic blood pressure (mmHg)	81 ± 8	82 ± 9	76 ± 12	76 ± 10	78 ± 10	83 ± 14
Primary Renal Diagnosis, *n* (%)						
Glomerular disease	5	5	3	4	2	3
Tubulointerstitial disease	2	3	1	3	1	2
Ischaemic nephropathy	2	5	2	5	2	4
Fibrosis score at baseline (*n*)	4(5), 3(2), 2(1), 0(1)	4(6), 3(3), 2(2), 0(2)	4(3), 3(1), 2(1), 0(1)	4(6), 3(2), 2(2), 0(2)	4(3), 3(1), 2(1)	4(4), 3(2), 2(2), 0(2)

## Data Availability

The data presented in this study are available on request from the corresponding author. The data are not publicly available due to ethical issues.

## Supplementary Materials

The following supporting information can be downloaded at: https://www.mdpi.com/article/10.3390/jcm12237282/s1, Figure S1. Bar charts showing the percentage change from baseline in MRI measures at Year 1 and Year 2 separated into progressors and stable groups for all participants recruited at baseline (*n* = 22) and those who remained in the study at Year 1 (*n* = 18) and Year 2 (*n* = 15).
